# A Novel Approach for Mapping Wheat Areas Using High Resolution Sentinel-2 Images

**DOI:** 10.3390/s18072089

**Published:** 2018-06-29

**Authors:** Ali Nasrallah, Nicolas Baghdadi, Mario Mhawej, Ghaleb Faour, Talal Darwish, Hatem Belhouchette, Salem Darwich

**Affiliations:** 1 IRSTEA, University of Montpellier, TETIS, 34090 Montpellier, France; nicolas.baghdadi@teledetection.fr; 2 National Center for Remote Sensing, National Council for Scientific Research (CNRS), Riad al Soloh, Beirut 1107 2260, Lebanon; mario.mhawej@gmail.com (M.M.); gfaour@cnrs.edu.lb (G.F.); tdarwich@cnrs.edu.lb (T.D.); 3 CIHEAM-IAMM, UMR-System, 34090 Montpellier, France; belhouchette@iamm.fr; 4 Faculty of Agriculture, Lebanese University, Beirut 99, Lebanon; salem.darwich@ul.edu.lb

**Keywords:** wheat, crop classification, Sentinel-2, NDVI, tree-like approach, Lebanon

## Abstract

Global wheat production reached 754.8 million tons in 2017, according to the FAO database. While wheat is considered as a staple food for many populations across the globe, mapping wheat could be an effective tool to achieve the SDG2 sustainable development goal—End Hunger and Secure Food Security. In Lebanon, this crop is supported financially, and sometimes technically, by the Lebanese government. However, there is a lack of statistical databases, at both national and regional scales, as well as critical information much needed in the subsidy and compensation system. In this context, this study proposes an innovative approach, named Simple and Effective Wheat Mapping Approach (*SEWMA*), to map the winter wheat areas grown in the Bekaa plain, the primary wheat production area in Lebanon, in the years of 2016 and 2017. The proposed methodology is a tree-like approach relying on the Normalized Difference Vegetation Index (NDVI) values of four-month period that coincides with several phenological stages of wheat (i.e., tillering, stem extension, heading, flowering and ripening). The usage of the freely available Sentinel-2 imageries, with a high spatial (10 m) and temporal (5 days) resolutions, was necessary, particularly due to the small sized and overlapped plots encountered in the study area. Concerning the wheat areas, results show that there was a decrease from 11,063 ± 1309 ha in 2016 to 7605 ± 1184 in 2017. When *SEWMA* was applied using 2016 ground truth data, the overall accuracy reached 87.0% on 2017 data, whereas, when implemented using 2017 ground truth data, the overall accuracy was 82.6% on 2016 data. The novelty resides in executing early classification output (up to six weeks before harvest) as well as distinguishing wheat from other winter cereal crops with similar NDVI yearly profiles (i.e., barley and triticale). *SEWMA* offers a simple, yet effective and budget-saving approach providing early-season classification information, very crucial to decision support systems and the Lebanese government concerning, but not limited to, food production, trade, management and agricultural financial support.

## 1. Introduction

With the steady increase of population and food demands in Lebanon [1], particularly following the massive influx of Syrian refugees since 2011, land degradation and mismanagement threaten food security. The latter is jeopardized as well by the partial and intermittent agricultural census, held once every 5–8 years depending on field questionnaires and farmers’ estimations. Even the national land cover/land use map, which is updated approximately every five years, contains no crop-specific classification. However, according to assumptions in 2010, the winter wheat cereal, supported by the Lebanese government as a strategic crop for food security in the country, occupied around 44% of the total field crop-cultivated land [2]. 

A regularly updated agricultural map, beginning with the identification of wheat parcels through remote sensing imageries, is then highly crucial for the Lebanese state and national statistics. These crop maps can assist decision-makers and end-users in identifying the cropped areas, estimating biomass production, water productivity, irrigation needs and scheduling, as well as defining management strategies. But more importantly, deriving statistics for annual cash crops to support sustainable national food security policies is vital [3,4,5,6].

In this context, previous studies have focused on generating the annual reference temporal profile using diverse Vegetation Indices (VI), such as the commonly known Normalized Difference Vegetation Index (NDVI) [3,7,8,9]. Low-resolution sensors such as the Advanced Very High Resolution Radiometer (AVHRR), with a spatial resolution of 1 km, and the Moderate Resolution Imaging Spectroradiometer (MODIS), with a spatial resolution of 250 m, were largely used to classify several crops such as corn and soybeans [10,11]. Results showed high accuracy (~80%) in terms of differentiation between major crops’ type (e.g., rice, corn, millet and cotton) [10,12]. However, and due to the low spatial resolution sensors, many heterogeneous pixels were mixed—crop/non crop, irrigated/non-irrigated, and even between different crops’ type [7,13,14].

The usage of sensors with higher spatial and temporal resolutions was then needed. Both [15,16] applied a decision tree algorithm to Landsat-8 imageries, with a spatial resolution of 30 m. Their approaches yielded high accuracy in mapping the available main crops (i.e., wheat, alfalfa, barley rice, trees, vegetables and potato). Another study [17] has showed that the inclusion of Gaussian kernel soft classifier, with Euclidean Norm in Possibilistic c-Means (KPCM), has been more robust in identification of the wheat crop when using Landsat 8 imageries. As for the temporal data, imageries corresponding to tillering, stem extension, heading and ripening stages of wheat crop would be the best combination to reach a highly accurate classification [17].

Another study [18] has considered the usage of both optical (i.e., Landsat-8) and radar (i.e., Sentinel-1 SAR) satellite imageries to improve early crop type (i.e., sunflower, wheat/barley, corn, soybean, grassland, alfalfa, bare soil, rapeseed and no-crop) classification. The obvious reason of merging the two imageries’ types is to create a “weather-independent” methodology. The proposed approach showed that the Kappa value increased to 73%, from 66% and 69%, when using Sentinel-1 and Landsat-8 solely, respectively. In the same context, McNairn et al. [19] integrated both optical and Synthetic Aperture Radar (SAR) imageries. Results showed that SAR images alone were not enough to accurately map crops. When only one or two optical images are available, the addition of two SAR images will improve overall accuracies and will boost individual crop classification matching to reach at least 85%.

Through the first experience with Sentinel-2 data for crop and tree species classification in central Europe, Immitzer et al. [20] employed a supervised random forest classifier (RF). They successfully mapped six summer crop species (i.e., carrots, maize, soya, onions, sugar beet and sunflower), in addition to winter crops and bare soil in lower Austria, as well as seven different deciduous and coniferous trees in Germany. Cross-validated overall accuracies ranged between 65% (tree species) and 76% (crop types). However, the study has also revealed the great potential of the red-edge and shortwave infrared bands for mapping vegetation. 

As saving resources has been having great attention recently, some studies have focused on the cross-year validation. For instance, in the central United States, soybean and corn were mapped using Landsat imagery with cross year validation [21]. Results have showed an average overall accuracy of 82%. The proposed approach required several sets of input variables (i.e., traditional spectral features at imaging dates, phenological metrics derived from EVI time series, spectral features and vegetation indices interpolated at phenological transition dates, and accumulated temperature during phenological stages). Thus, with high complexity of data sources, the implementation of such approach could be challenging.

Early crop mapping has been the focus of recent work, especially when coupled with remotely-sensed data. In 2015, a study was conducted in France to assess the contribution of very high spatial resolution (VHSR) Pléiades images to early season crop identification. The validation of the approach showed a drop in overall accuracy from 79%, when considering winter cereals as a composite class, to 69% when discriminating among winter wheat and winter barley [22]. In a recent study [23], MODIS NDVI time-series data, crop mask and growing degree days were used to map winter crops in an automated way up to two months prior to harvesting period. Their results have showed accuracy exceeding 90%. While it is highly important to execute early season mapping, further crop-specific classification, with a high overall accuracy, is much needed at regional and national scale.

In the abovementioned studies several shortcomings were observed: (1) wheat classification could not be carried out before the end of the cropping season (i.e., during maturation stage); (2) results have not been validated on different cropping seasons; and (3) they did not distinguish among similar VI-annual profile cereal crops (e.g., barley and triticale). In this context, this paper will be examining the ability of the new high resolution optical sensor Sentinel-2 with 10 m spatial and 5 days temporal resolutions, to accurately map winter wheat in the Bekaa plain of Lebanon using a novel, yet simple classification approach, named Simple and Effective Wheat Mapping Approach or *SEWMA*. It is a decision tree-like algorithm, based on the NDVI values that is able to overcome the major challenges of achieving high accuracy classification before the end of the cropping cycle, could be portable to other years, and can distinguish wheat from barley and triticale. The implementation of *SEWMA* approach at regional/national scale shall enable an adequate planning and managements by decision makers and governments while saving on resources and monetary values for field based statistics. Section 2 presents the study site and the cropping calendar, followed by Section 3 which describes the database and materials used. Section 4 describes the methodology of the proposed approach. The results are shown in Section 4. Section 5 presents a discussion of the important results, followed by a conclusion.

## 2. Study Area

**Figure 1:**
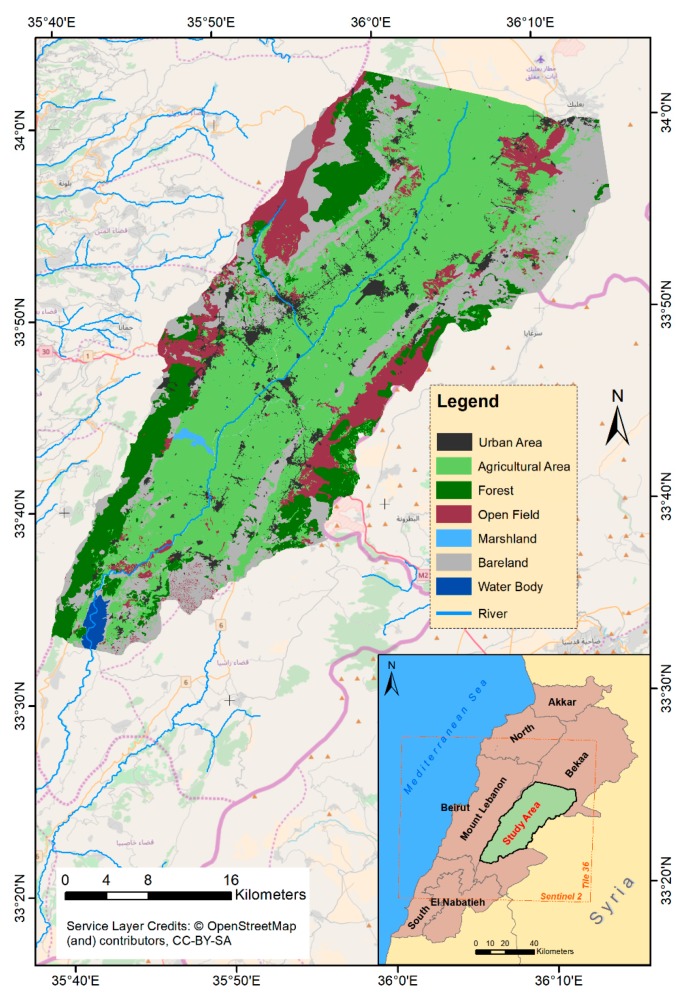
Location of Bekaa plain of Lebanon as well as Sentinel-2 (in orange) tile covering the study area (Landcover/Landuse NCRS-L, 2013).

The selected study area, the Bekaa plain, is located between 33°33′ N and 33°60′ N latitude, 35°39′ E and 36°14′ E longitude (Figure 1), covering an area of 860.25 km^2^. The plain lies between two natural units having very steep slopes; the eastern slopes of the Mount-Lebanon Mountains (western unit) and the Western slopes of the Anti-Lebanon Mountains (eastern unit). The average elevation of the study area is around 1000 m above sea level (a.s.l.). The study area is characterized by a semi-arid (northern part) and dry-Mediterranean (southern part) climate and the average annual precipitation is around 600 mm [24].

**Figure 2:**
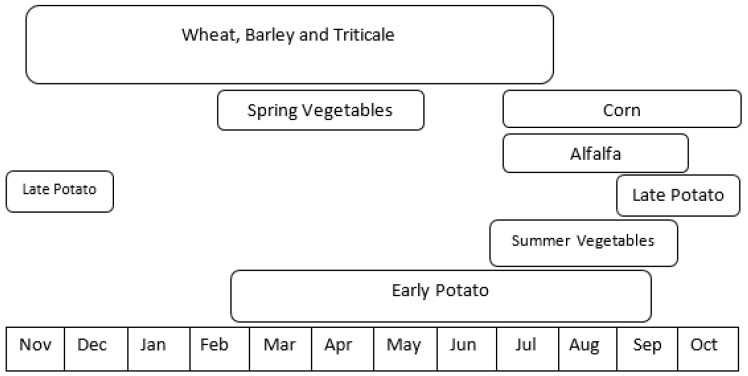
Different crops calendars at the Bekaa plain (adopted from USAID [25]).

Agriculture is the main economic activity in the Bekaa plain, including several field crops (e.g., wheat, potato, barley and alfalfa) of various field areas ranging from 0.1 ha to more than 20 ha. However, the wheat parcels predominate in the areas, corresponding to more than 65% of national cereal production [2]. Wheat, as well as the other local cereals (i.e., barley and triticale), have a very similar phenological cycle as they are sown in November and harvested next year in June. In addition to the cereals, other spring and summer crops (e.g., potato, corn, vegetables and alfalfa), are being cultivated in the Bekaa plain. Figure 2 illustrates the crop calendar of the main field crops grown in the plain. 

## 3. Material and Methods

### 3.1. Datasets and Preprocessing

**Figure 3:**
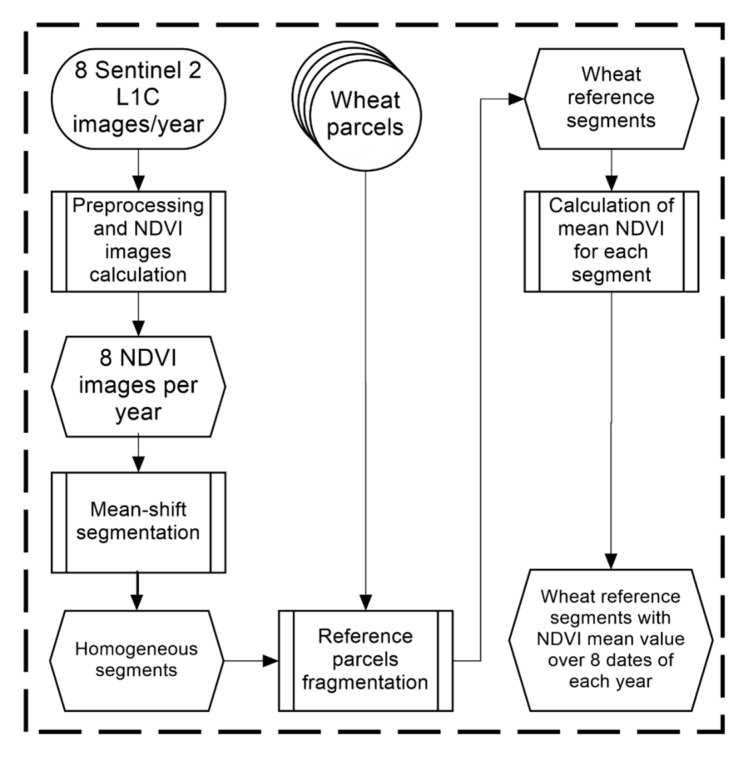
Simplified flowchart for the preparation of SEWMA NDVI temporal profiles.

Two types of datasets were essential to conduct this study: two-year field data containing ground reference plots to train and validate our approach, and corresponding Sentinel-2 imageries (each tile is of 100 × 100 km^2^). These datasets were used to extract the NDVI temporal profile, as it was the main key to eventually classify winter wheat at the Bekaa plain of Lebanon during the two years of study (i.e., 2016 and 2017). Figure 3 represents a flowchart summarizing the preparation work, whose output will be used as input for our proposed approach.

#### 3.1.1. Satellite Data

Sentinel-2 is the second generation Earth Observation (EO) satellite operated by the European Space Agency (ESA) [26]. The launching of Sentinel-2A and Sentinel-2B was in June 2015 and March 2017 respectively, as an integral part of Europe’s Copernicus program aiming at independent and continued global observation capacities [20]. Sentinel-2 offers a fine spectral, spatial and temporal resolutions (i.e., 13 bands ranging from 10 m to 60 m with a revisit time of five days). Datasets produced by this satellite could be downloaded free of charge from Europe’s Copernicus website [27]. Eight Sentinel-2 images were used each year (i.e., 2016 and 2017) between January and May (Table 1), as these images cover the main phenological stages. The pre-processing of L1C (Top of Atmosphere or TOA reflectance) Sentinel-2 images, which includes ortho-rectification, cloud removal (using cloud mask produced by Sen2Cor/SNAP), radiometric calibration and atmospheric correction, was produced using SNAP/Sentinel-2 toolbox. The output of the pre-processing, corresponds to L2A (Bottom of Atmosphere or BOA reflectance).

The Normalized Difference Vegetation Index (NDVI), which ranges from −1 to 1 is successful in predicting photosynthetic activity as it is computed from the Red (*ρ*RED) and Near Infrared (*ρ*NIR) reflectance values, corresponding to Bands 4 and 8, respectively, as follows:

(1) $$\mathrm{NDVI}=\frac{\rho \mathrm{NIR}-\rho \mathrm{RED}}{\rho \mathrm{NIR}+\rho \mathrm{RED}}$$

There is a strong correlation between the NDVI ratio and above ground green biomass [28]. In other words, as green biomass increases, NDVI reflectance tends to get closer to 1, thus, spectral measurements are strongly related to the amount of leafy biomass [29,30].

As in our case, during winter, the wheat canopy tends to go through a dormancy stage where development is paused until reaching a certain Growth Degree Days [31]. During this period, NDVI normally does not exceed 0.3. After stem elongation and booting stage are initiated, NDVI comes closer to 1 [32].

While applying the mean shift segmentation (post cloud removal) to the study area for each year, the area of interest was clustered into homogeneous units (segments) in each year (2016 and 2017). For both years, eight NDVI images (Table 1) were stacked together and used as an input to the mean-shift algorithm, to produce unique homogeneous units’ map (segments) for each year (i.e., 2016 and 2017). The mean-shift segmentation [33,34] is a widely used segmentation approach [35], firstly proposed by [36]. It relies basically on spatial and range radii and was executed in this paper using the open source software QGIS [37]. The segmentation parameters are: Spatial radius = 10 pixels and NDVI range radius = 0.1. The reason behind setting such parameters is, first, since the resolution of Sentine-2 is 10 m, then ten pixels are 1000 m^2^, which is the minimum cultivated area by farmers in the studied region; second, since the variability in NDVI within our reference plots over the eight dates used did not exceed 0.1 as NDVI value, a 0.1 range radius was used.

#### 3.1.2. Ground Data

Field visits were carried out between February and June of the corresponding years (i.e., 2016 and 2017), as this period covers the most critical wheat phenological stages needed for classification. Cereals plots (i.e., wheat, barley and triticale) as well as other cultivated plots (i.e., spring potato and spring vegetables, fruit trees, vineyards, and alfalfa) and bare soil areas were visited and their coordinates were recorded as reference plots (Table 2). These plots were fragmented according to the segmentation output (produced earlier) of each year and used for training and validation processes.

#### 3.1.3. Temporal Profile Analysis

In order to produce the NDVI-temporal profiles for the main cultivations in the study area, the mean and the standard deviation of the NDVI images were calculated at segment level.

The behaviors of the reflectance of the main cereals (i.e., wheat, barley and triticale), spring potato and spring vegetables in the NIR, Red and NDVI are presented in the Results section (Section 4.1).

### 3.2. SEWMA Generation

The main objective of this study is to map the spatial distribution of the wheat segments four to six weeks prior to the harvesting period for both 2016 and 2017 cropping seasons. The methodology proposed consists basically of two phases. The first phase discriminates wheat candidate segments (plots or sub-plots), which could be wheat, barley or triticale plantation, from other land-cover types. For this purpose, we extracted the NDVI temporal profile from the Sentinel-2 imageries for the three winter cereal crops (i.e., wheat, barley and triticale). Using the wheat NDVI values of each date of the eight dates, linear relationships were established between each date and the date that follows. The parameters resulted from those linear relationships were used to simulate NDVI images that allowed further to select wheat candidate segments (end of the first phase).

By referring to the NDVI temporal profile of the three winter cereal crops and following the application of several conditions, the second phase enables the selection of wheat segments from the others plantations (i.e., barley and triticale).

**Figure 4:**
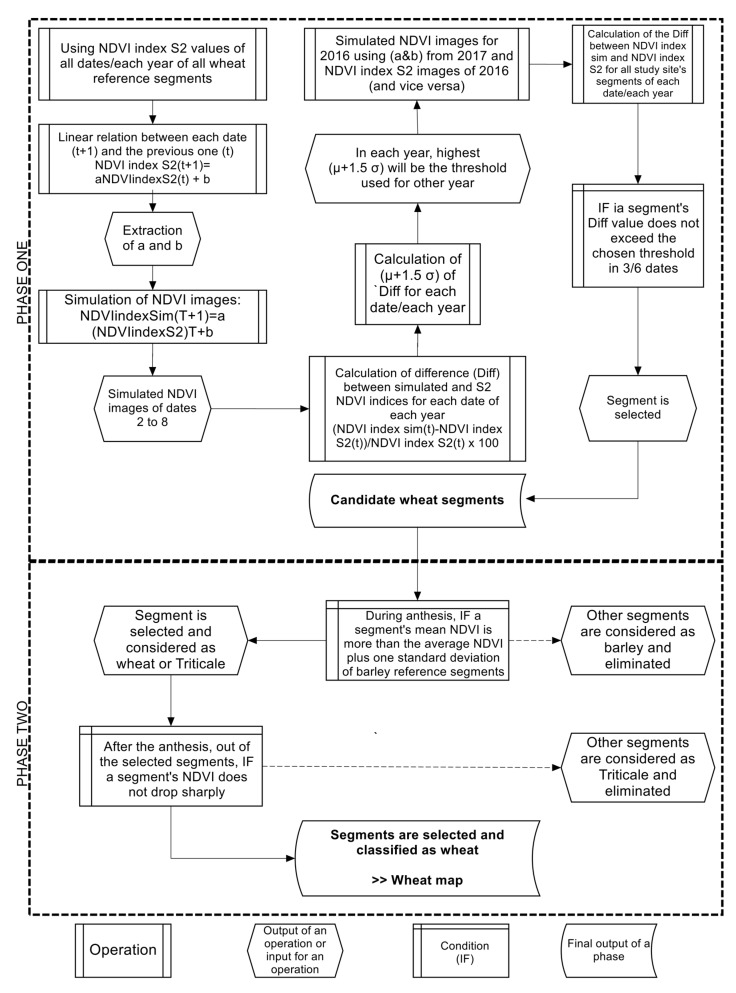
*SEWMA* (Simple and Effective Wheat Mapping Approach) simplified flowchart.

As we intended to train and validate *SEWMA* using different years, all reference wheat segments collected (Table 2) were used for the training and validation processes. When *SEWMA* was trained with 2016 reference segments, the application was on 2017 Sentinel-2 (S2) images, and when trained with 2017 reference segments, the application was on 2016 Sentinel-2 (S2) images. Thus, 348 wheat segments were used to train year 2016, and 216 wheat segments were used to train year 2017. In the coming sections, we will be presenting a detailed description of each phase. A simplified flowchart of the methodology is illustrated in Figure 4 below.

#### 3.2.1. Identification of Wheat Candidate Segments: First Phase

Using the Sentinel-2 mean NDVI per wheat segment values, identified from the field campaigns (reference wheat segments) in 2016 and 2017, linear relationships of their NDVI values were established for each year between each two consecutive dates. In this study, we have used linear fitting because of the short-term data used. Moreover, in terms of NDVI real value, the development of wheat has proven to be predicted between two dates (*t* and *t* + 1) in a linear manner. This predictability was particularly essential because different wheat plots present different NDVI values through their development. With the usage of these relationships on different dates, segments could be eventually selected as wheat candidate segments (i.e., wheat, barley or triticale). The reference wheat NDVI linear relationship between each two consecutive dates (*t* and *t* + 1) is defined by:

(2) $$NDVI_{S2}\left(t+1\right)=a\times NDVI_{S2}\left(t\right)+b$$

By using the linear relationships (slopes “a” and interceptions “b”), we simulate NDVI images $\left(NDVI_{Sim}\right)$ for each date of the Sentinel-2 $\left(NDVI_{S2}\right)$ images. The slopes and interceptions deduced from the already produced linear relationships for each date used, are listed in the Results (Section 4.2).

To simulate NDVI images for the dates of 2016, “a” and “b” coefficients (Section 4.2) deduced from linear relationships (Equation (2)) of 2017 Sentinel-2 NDVI images were used, in addition to the Sentinel-2 NDVI images of 2016. Same is applied when simulating NDVI images for the dates of 2017. For each wheat reference segment, the following equation is applied:

(3) $$NDVI_{Sim}\left(t+1\right)=a\times NDVI_{S2}\left(t\right)+b$$

However, with a minimum coefficient of determination (R^2^) of 0.52, the application of these linear relationships could under- or over-estimate the probability of a segment being a candidate wheat plantation. In this context, the addition of a margin of error is required. After the production of the simulated NDVI images $\left(NDVI_{Sim}\right)$, we calculate the average Sentinel-2 NDVI $\left(NDVI_{S2}\right)$ as well as the average simulated NDVI $\left(NDVI_{Sim}\right)$ for each reference segment of each date in both years of study. Then, the difference between the average simulated NDVI $\left(NDVI_{Sim}\right)$ value and the average Sentinel-2 NDVI $\left(NDVI_{S2}\right)$ value was calculated for each reference segment in all dates of both years.

For each reference segment, the difference (Diff) is produced between simulated NDVI and S2 NDVI values, in each date of each year, as follows:

(4) $$Diff\left(t\right)=\left[\frac{NDVI_{Sim}\left(t\right)-NDVI_{S2}\left(t\right)}{NDVI_{S2}\left(t\right)}\right]\times 100$$

After the calculation of the differences between simulated NDVI and Sentinel-2 NDVI of each reference segment in each date of each year, we calculate both the average μ(*t*) and the standard deviation σ(*t*) of the obtained differences, for each date. Using μ(*t*) and σ(*t*) of the differences among the dates, and to ensure the highest accuracy with least over- and under-estimations, three thresholds were selected as follow: (1) [μ + *1*σ]; (2) [μ + *1.5*σ]; and (3) [μ + *2*σ], for each year. These thresholds are seen as the margin of errors used. The difference of wheat reference segments when using the three thresholds for both years are illustrated in box plots in the Results (Section 4.2). The most adequate threshold should have the highest accuracy in determining the wheat segments. It will be defined following the implementation of *SEWMA* (Section 4.3) through the production of confusion matrices for each selected threshold. Nonetheless, for now, these three thresholds should be used.

In each year (2016 and 2017), the highest [μ(*t*) + *n*σ(*t*)] value among the dates was chosen to represent the threshold of its corresponding year, where *n* is either 1, 1.5 or 2 depending on the chosen threshold. For instance, when using the threshold μ(*t*) + *1.5*σ(*t*), its value on date 4 in 2016 (6th of April) was the highest (27%), thus, 27% was assigned as a threshold produced by 2016 wheat reference segments. A criterion now has to be met; at least in three out of the first six dates (DOY 47 through 137 for 2016 and DOY 41 through 131 for 2017), segmented areas should have a difference (Diff) between simulated NDVI and S2 NDVI within the chosen threshold. If so, the segment is then considered as potential wheat cultivation. The later could also pinpoint at barley or triticale segments. The other segments are eliminated and not considered in the further processing steps. As a result, the output of the first phase is the identification of wheat candidate segments.

#### 3.2.2. Identification of Wheat Segments: Second Phase

By referring to the NDVI temporal profile analysis (Section 4.1), it was found that barley can be distinguished from wheat on DOY 107 through 117 in 2016 and DOY 131 in 2017. While these periods generally highlight the anthesis of wheat, the NDVI average value for barley segments is generally lower than the NDVI average value for wheat cultivation. The difference between barley and wheat in terms of mean NDVI could be justified in terms of water availability/uptake since wheat is supplementary irrigated during the season, whereas, barley is generally a rain-fed cultivation [38].

If a segment’s mean NDVI is less than the total average NDVI plus the standard deviation of barley reference segments in anthesis, then it is considered as barley plantation and thus eliminated. As for triticale, and by consulting the NDVI temporal profile analysis (Section 4.1), on DOY 137 in 2016 and 151 in 2017, a huge drop of NDVI average value is shown. While these dates correspond to the harvesting period of triticale, thus rendering lands without vegetation cover and very low NDVI, the NDVI values for triticale plots are generally much lower than the NDVI values for wheat cultivation. Accordingly, if the difference between the date corresponding to anthesis and the date afterwards (*t* + 1) is lower than 70% (value set upon our observations on 645 wheat and triticale reference segments), at segment level, then this segment is classified as wheat. The others reflect triticale cultivated segments and thus eliminated. Therefore, the output of the second phase is the identification of wheat segments relying on the NDVI real values. In that event, after selecting the candidate segments (at the end of phase one) and the elimination of barley and triticale segments (at the end of phase two), wheat segments are then identified for both 2016 and 2017 years. It is important to note that the proposed approach was established by 2016 datasets and validated through 2017 datasets, and vice versa, using three thresholds (i.e., (1) μ + *1*σ; (2) μ + *1.5*σ; and (3) μ + *2*σ) as mentioned above.

#### 3.2.3. Validation

Accuracy assessments were done to evaluate the classification approach presented in this study. Since the classification approach for 2016 was done by relying on 2017 ground truth data (GTD) to calibrate it, and vice versa, we had to test the overall accuracy of 2016 classification using 2016 GTD and for 2017 using 2017 GTD. For this purpose, wheat segments collected from 2016 were used to validate year 2016, and wheat segments collected in 2017 were used to validate year 2017. As to run the confusion matrix, wheat and non-wheat segments were needed. Same number as wheat segments was chosen for non-wheat segments. For 2017 classification, we had 348 wheat segments, thus an equivalent number of non-wheat segments were randomly chosen from the whole area of study so that the total number of segments to run the confusion matrix of 2017 was 696 consisting of 50% wheat segments and 50% non-wheat segments. Same for 2016, the total number of segments used was 432, consisting of 50% wheat segments and 50% non-wheat segments.

## 4. Results

In this section, we report the results of the proposed method. The obtained temporal profiles of the crops analyzed are presented in this section (Section 4.1). In addition, the preliminary results deduced from the first phase of *SEWMA* generation are also illustrated (Section 4.2). The accuracy assessment is reported for both years (Section 4.3) as well as the spatial distribution of wheat plots (Section 4.4).

### 4.1. Crops’ Temporal Profiles

**Figure 5:**
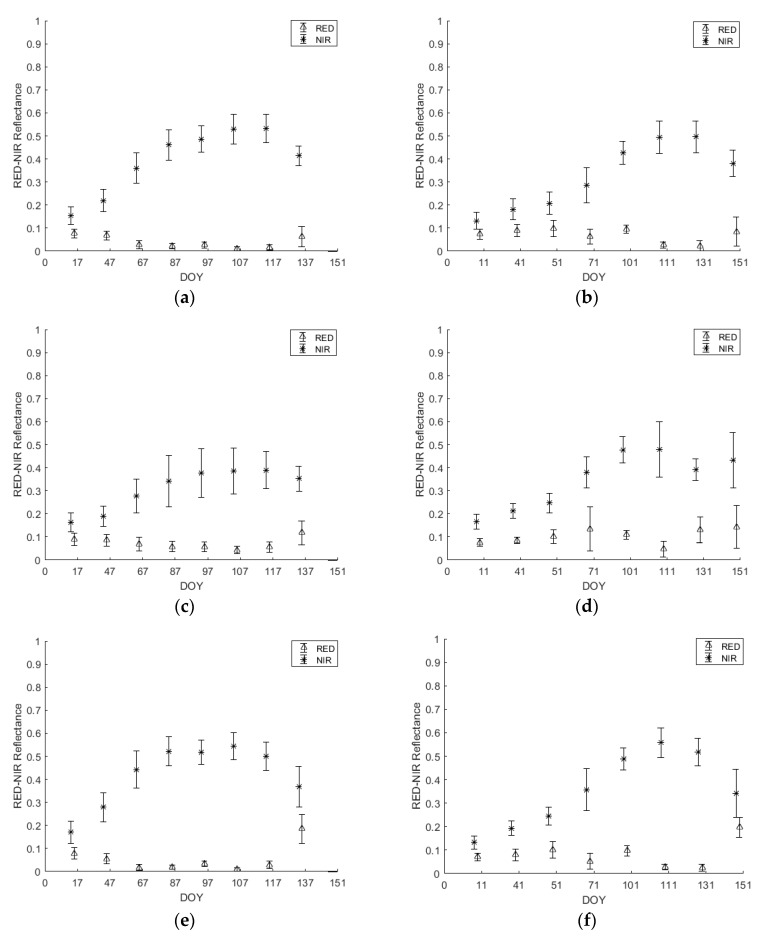
Mean ± standard deviation of *ρ*RED and *ρ*NIR temporal profiles of Wheat (**a**) 2016 and (**b**) 2017; Barley (**c**) 2016 and (**d**) 2017 and Triticale (**e**) 2016 and (**f**) 2017.

**Figure 6:**
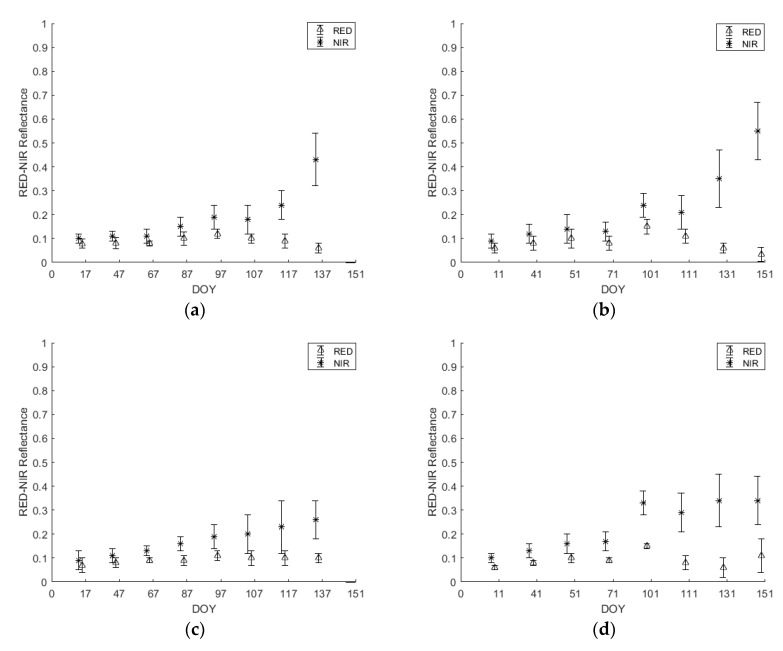
Mean ± standard deviation of *ρ*RED and *ρ*NIR temporal profiles of spring potato (**a**) 2016 and (**b**) 2017 and spring vegetables (**c**) 2016 and (**d**) 2017.

Figure 5 and Figure 6 represent the mean ± standard deviation of *ρ*NIR and *ρ*RED temporal profiles. The mean and the standard deviation values for the crops below (Figure 5 and Figure 6) were extracted from the Sentinel-2 images for each date in each year (i.e., 2016 and 2017).

By analyzing the differences among years, up to the third date, corresponding to heading stage (DOY 97/2016 and 101/2017), the three cereal cultivars (i.e., wheat, barley and triticale) have higher reflectance in the NIR band in 2016 than 2017 and lower reflectance in the RED band in 2016 than 2017 (Figure 5). Among crops, wheat and triticale experienced a very similar behavior in terms of reflectance in both bands and years until the date when triticale was harvested (DOY 137/2016 and 151/2017). Whereas for barley, the reflectance in the NIR band was significantly lower than that of wheat and triticale for DOY 107-117/2016 and 131/2017, and the reflectance in the RED band was significantly higher than the reflectance in the RED band for wheat and triticale for DOY 131/2017.

In order to make sure there was no classification confusion between the three main cereal crops (wheat, barley and triticale) and other crops that share part of their seasons, *ρ*NIR and *ρ*RED were analyzed for spring potato and spring vegetables (Figure 6).

As the sowing dates of spring potato and spring vegetables do not occur before March, the reflectance values in the RED and NIR bands were close to each other (Figure 6). For spring potato, after the sowing date in March, the reflectance in the NIR began to increase (DOY 87/2016 and 101/2017) reaching the peak of around 0.55 in DOY 137/2016 and 0.65 in DOY 151/2017. Nevertheless, the reflectance in the RED band also started experiencing a change and decreased to reach its minimum of around 0.04 in DOY 137/2016 and 151/2017 corresponding to the potato full flowering stage.

As for spring vegetables, reflectance in the RED and NIR did not start to change before DOY 97/2016 and 101/2017 (Figure 6). For the reflectance in the NIR band, the maximum was reached in DOY 137/2016 (0.35) and DOY 151/2017 (0.42). As for the reflectance in the RED, the minimum was reached in the same dates of 0.07 and 0.03 in 2016 and 2017 respectively. 

**Figure 7:**
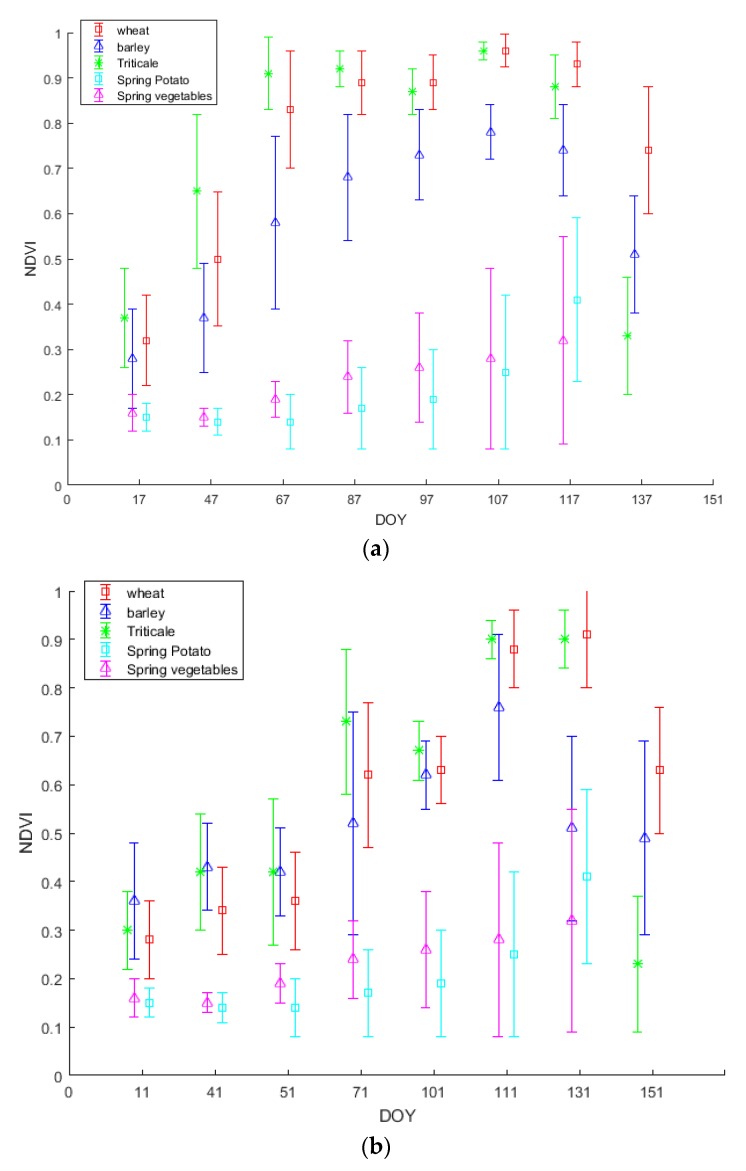
NDVI temporal profile of wheat, barley, triticale, spring potato and spring vegetables of 2016 (**a**) and 2017 (**b**) years.

The time profile of the NDVI with respect to time (dates of the available images) was constructed and analyzed for the three main cereal crops (i.e., wheat, barley and triticale) in addition to spring potato and spring vegetables during the two years of study (2016 and 2017). The evolution of NDVI along both cropping seasons for the three main crops is shown in Figure 7, representing the mean ± standard deviation of NDVI temporal profiles.

Figure 7 clearly shows that the NDVI behavior during 2016 and 2017 of the cereal crops is not the same throughout the cropping season. The temporal profiles of the three main cereal classes (wheat, barley and triticale) during both years (Figure 7a,b) show the crop evolution after emergence through maturity (wheat and barley) and harvesting (triticale). In 2016 (Figure 7a), the three classes finished the vegetative growth and reached anthesis in DOY 107. After that, wheat and barley started their maturation stage in DOY 137 while triticale was already harvested to be sold for fodder use. In 2017 (Figure 7b), the dormancy stage was relatively longer than 2016 (Figure 7a), which led to a different temporal profile, and anthesis was reached between DOY 111 and DOY 131. According to the NDVI images corresponding to the anthesis stage of both years, barley’s NDVI was significantly lower than wheat’s NDVI. As for spring potato and spring vegetables, indeed these two classes share some of their crop cycle with the three main cereal crops. As they are sowed between end of February and start of March, they start to witness an increase in their NDVI starting DOY 97 in 2016 and DOY 101 in 2017, thus, they are easily separated and do not actually interfere in our classification of winter wheat.

### 4.2. SEWMA First Phase Preliminary Results

Through the first phase of *SEWMA* generation, linear relationships of wheat reference segments’ NDVI values were established for each year between each two consecutive dates (Equation (2)). The output parameters (slopes “a” and interceptions “b”) are listed in Table 3, which were used to simulate NDVI images (Section 3.2.1) using Equation (3).

**Figure 8:**
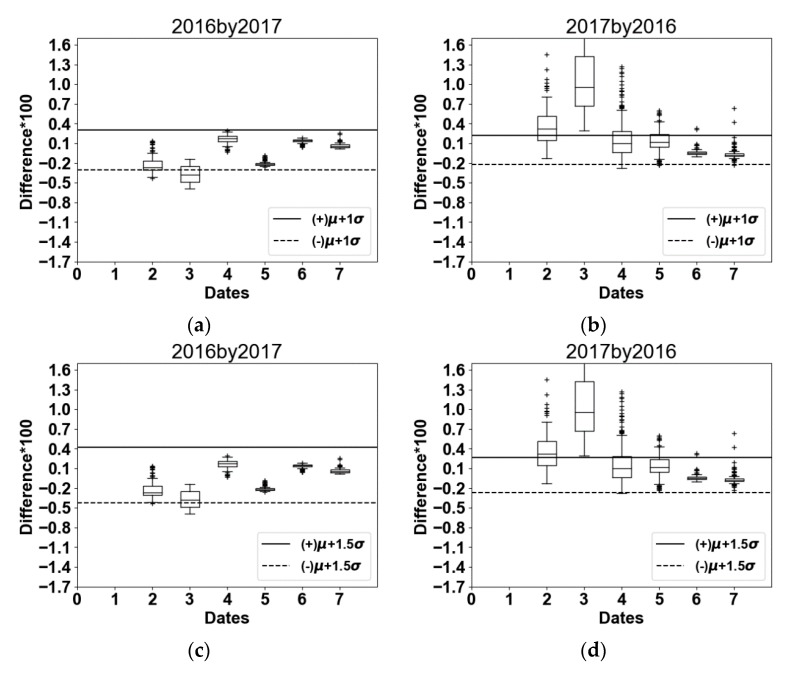

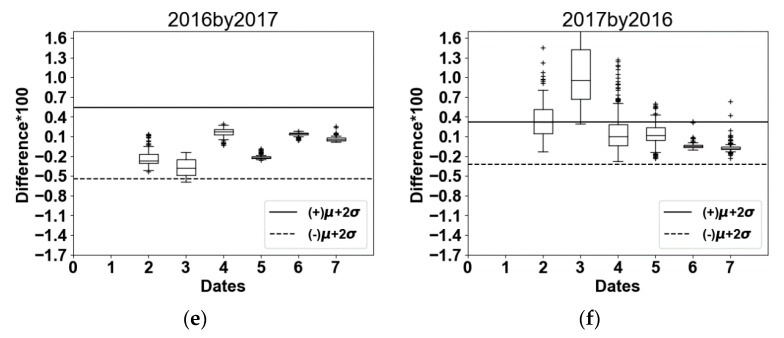
Differences of wheat reference segments when using the thresholds [μ + *1*σ] (**a**) 2016 when calibrated by 2017 and (**b**) 2017 when calibrated by 2016, [μ + *1.5*σ] (**c**) 2016 when calibrated by 2017 and (**d**) 2017 when calibrated by 2016 and [μ + *2*σ] (**e**) 2016 when calibrated by 2017 and (**f**) 2017 when calibrated by 2016.

Through the identification of wheat candidate segments (Section 3.2.1), and after NDVI images were simulated depending on the parameters presented in Table 3 above, the differences between Sentinel-2 and simulated NDVI values versus thresholds assigned are presented in Figure 8.

As (Diff) (Equation (4)) reflects the difference between simulated NDVI and Sentinel-2 NDVI, the differences of wheat reference segments (Figure 8) could be either positive or negative. For this reason, the threshold is expressed above positively and negatively.

### 4.3. SEWMA Accuracy Assesment

The approach accuracy assessment was produced for the three thresholds (i.e., μ + *1*σ, μ + *1.5*σ and μ + *2*σ) (Table 4). It clearly shows that the implementation of the 2016 approach on 2017 generates a higher accuracy from, inversely, applying the 2017 approach on 2016. Also, according to this same table, the best accuracy was noted in the second threshold used (i.e., μ + *1.5*σ).

A confusion matrix was presented for the second threshold (i.e., µ + *1.5*σ) (Table 5 and Table 6). By using the 2016 trained wheat approach classification on 2017, the overall accuracy reached 82.6%. When applying the 2017 trained wheat approach classification on 2016, the overall accuracy reached 87.0%.

### 4.4. Wheat Spatial Distribution

**Figure 9:**
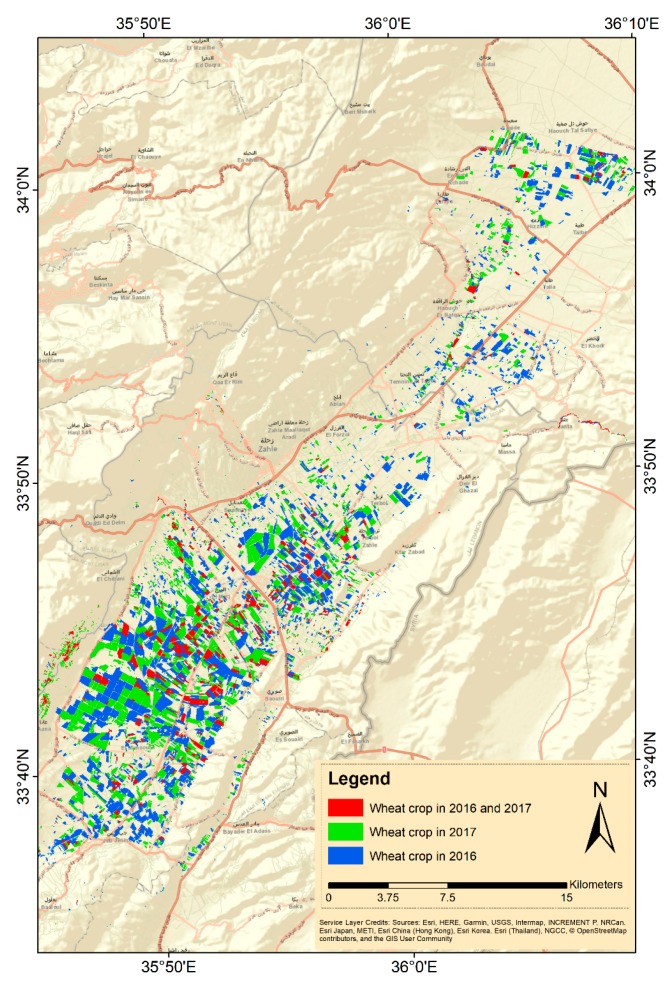
Spatial distribution of wheat in the Bekaa plain for years 2016 and 2017.

Table 7 shows the wheat cultivated areas in the Bekaa plain estimated based on the reference sample data, with 95% confidence interval according to [39], in addition to wheat areas declared by the Lebanese government. Wheat cultivated areas below present a decrease from 2016 to 2017. This area is densely distributed in the center and to the southern part of the study site (Figure 9). Crop rotation is also noticeable in comparison among the plots (Figure 9).

According to the wheat spatial distribution in Figure 9, cultivation of wheat was denser in the south west of the plain comparing to the northern part, as water is more available, thus more compatible to irrigation management. Rotation is also visible as most farmers follow the traditional potato-wheat rotation. However, A number of plots have witnessed wheat cultivation in the two consecutive years (2016 and 2017) occupying up to 28% of plots cultivated in monoculture each year.

## 5. Discussion

### 5.1. Crops’ Temporal Profiles

Winter wheat, which was classified in this study, is sown in November, similar to other winter cereals (i.e., barley and triticale). The winter cereals (i.e., wheat, barley and triticale) go through successive phenological stages during the cropping season, which are reflected in the NDVI temporal profiles.

In each year (Figure 7), the winter cereals (i.e., wheat, barley and triticale) showed similar evolution in terms of NDVI until the vegetative growth was almost over (anthesis period), where the peak NDVI value is reached. Generally, irrigated crops have been found to have a higher peak NDVI values and maintain a higher NDVI during each crop’s growth cycle than non-irrigated crops [13]. By the end of March, one supplementary irrigation had been already applied earlier that month to wheat and triticale. Due to this, wheat and triticale’s NDVI values rise to get closest to 1, whereas barleys’ NDVI values become significantly separable. By referring to Figure 5, this finding was reflected in the NIR and Red bands results. On DOY 107 through 117, barley exhibited a significant lower reflectance in the NIR band than wheat and triticale and a slightly higher reflectance in the visible (Red) band. As previously mentioned, the less leaf water content in barley than wheat and triticale could be responsible for such a drop [40].

After the flowering period, when triticale reaches maximum vegetative growth, farmers rush to harvest the triticale-cultivated plots before maturation kicks in. For this reason, triticale plots witness a sharp drop in their NDVI values due to harvesting event, thus triticale becomes significantly distinguishable. On further justification to such finding in the NDVI temporal profile (Figure 7), referring to Figure 5e,f, when harvesting triticale, the gap between the reflectance of Red and NIR bands is minimized. On one hand, the reflectance in the NIR decreased as the green cover is cut, thus the leaf water content is diminished, while on the other hand, the reflectance in the visible Red band increased because the contribution of chlorophyll pigments in the absorption in the Red band is significantly reduced, due to the harvesting event [29,41].

As for the other analyzed crops (i.e., spring vegetables and spring potato), their sowing date is in March. Spring vegetables and spring potato start their vegetative development in spring, yet their NDVI does not reach high levels as they do not fully cover the soil. According to the NDVI temporal profiles (Figure 7), spring vegetables and spring potato could be significantly separable from the three winter cereals. Referring to Figure 6 can explain such response. The increase in NDVI (Figure 7) during the spring is related to the increase in NIR reflectance (due to increase in leaf water content), and the decrease in the visible Red reflectance (due to the increase in leaf chlorophyll pigments) (Figure 6) [41].

The standard deviation of the winter cereals was higher in the beginning of the season, probably due to different germination rates [29] and decreased gradually through reaching the anthesis stage. When anthesis stage is reached, the canopies reach their maximum height as vegetative growth stops when flowering occurs [29]. By the end of the vegetative growth, maximum leaf area is reached, which was reflected in the NDVI temporal profile [29,42]. This difference in germination rates could be due to sowing date [43], variation in soil conditions [44], wheat varieties [45] and/or climate [46]. For spring vegetables and spring potato, during the first three dates, the standard deviation was low as the crops were not germinated before April. After germination, the standard deviation increased as different varieties of different crops were grouped together [45].

According to the Lebanese Agricultural Research Institute (LARI), both years were climatically different as winter season in 2017 was colder than 2016. This was obviously reflected in the NDVI temporal profiles. In 2017, by the end of February, the winter cereals’ NDVI had not reached 0.6, whereas by that time in 2016, winter cereals’ NDVI have had reached higher values already (Figure 7). Such response in the NDVI reflectance is basically related to the dormancy period [47] which was relatively shorter in 2016 than in 2017.

### 5.2. SEWMA First Phase Preliminary Results

In the proposed approach, we did use linear fitting between each two adjacent dates (i.e., *t* and *t* + 1). The linear fitting between each two consecutive dates produced slopes and interceptions (Table 3), which were used afterwards to simulate NDVI images. Due to variability in weather conditions and imaging dates, curve-fittings classifiers cannot be trained and applied on different years [48]. As *SEWMA* was built using short-term data, linear fitting was used rather than harmonic analysis, as the latter is suggested, when detecting changes in land use/land cover over a period of years is necessary [49].

In addition, smoothed temporal profile (e.g., moving window method) used in curve-fittings [21] might produce a curve that do not represent only wheat plots, but also other cultivations. Moreover, we did find that a date (*t* + 1) could be predicted from date (*t*), which reflects that the development of the wheat could be predictable with high accuracy in different wheat parcels and in diverse climate and regions presented in our study area.

Predicting NDVI*_t_*_+1_ from NDVI*_t_* could under- or over-estimate the probability of a segment being a candidate wheat plantation. In this context, the addition of a margin of error was required, by setting thresholds (Figure 8), which could be avoided when using curve-fitting techniques [48]. The choice of the selected threshold is discussed in the following section (Section 5.3).

### 5.3. SEWMA Accuracy Assessment

*SEWMA* was run in parallel using the three thresholds (i.e., μ + *1*σ, μ + *1.5*σ and μ + *2*σ) and μ + *1.5*σ showed the highest final overall accuracy (Table 4), thus μ + *1.5*σ was adopted. μ + *1.5*σ threshold allowed us to select the wheat candidate segments (after the first phase) with less under estimation than μ + *1*σ and less over estimation than μ + *2*σ. 

The underestimation of wheat classification that occurred when applying 2016 linear relationships on 2017 was mainly for two reasons. First, since the threshold was produced by 2016 ground truth data, few wheat segments did not cross the first phase of the approach, as they exceeded the threshold set in more than 3 dates. The difference in climate among the two years (2016 and 2017) was reflected via the NDVI profiles, hence these few segments were not considered as candidate segments and eliminated after the first phase. Second, during the second phase of the approach, the mean NDVI of reference barley segments plus the standard deviation at the anthesis period (DOY 117 of 2016) used to designate barley segments, was around 0.84 (Figure 7a). Thereby, some wheat segments were removed. This elimination is mainly related to the fact that the year 2017 was a cold and wet year, and since the season of wheat was longer than that in 2016, the NDVI of some wheat segments in 2017 was lower than that in 2016 during anthesis (DOY 117/2016 and 131/2017).

As shown in Figure 8d (2017 by 2016), since the dormancy period was longer in 2017 than 2016, one date (date 3) was completely out of the threshold borders. In date 3/2017 wheat was still through the dormancy stage (cumulative Growth Degree Days did not exceed 300 °C) and the NDVI did not exceed 0.45, while in date 3 of 2016, wheat’s NDVI has had reached 0.9 already (cumulative Growth Degree Days exceeded 450 °C) [30]. If unlike our case, both years were climatically similar, less wheat segments will be eliminated after the first phase of the approach.

When the approach was trained by 2017 ground truth data, the overall accuracy showed 82.6% when validated on 2016 images. The decrease in the overall accuracy was basically due to selecting some barley segments as wheat. This is because the classification was trained by 2017 GTD. As we refer to Figure 7a, in DOY 117, some barley segments had NDVI above 0.8.

Winter wheat plantations at the Bekaa plain receive some supplementary irrigation during the spring-early summer season, hence their NDVI reaches higher level than barley during the anthesis period. However, if some growers cultivate wheat with no supplementary irrigation (due to water shortage), such wheat segments would have similar NDVI values as barley and could be eliminated through phase two of *SEWMA*.

Our overall accuracies were satisfactory, similar to other previous studies, aiming at mapping winter crops [13,50,51], especially early-season classification [22,23]. Discriminating winter wheat from other winter cereal crops especially barley, as proposed by our approach is highly challenging, as it was shown in a previous study, where accuracy dropped to below 70% when cereals were ungrouped and winter wheat was discriminated from barley [22].

The difference in accuracy among both years (i.e., 2016 and 2017) is attributable to several reasons: (1) different number of training segments (plots or sub-plots); (2) different climatic conditions among the two years and (3) slightly different shift in the dates of available Sentinel-2 images.

### 5.4. Wheat Spatial Distribution

As wheat growth, tillering, biomass and grain yield are highly affected by soil moisture [52,53], the dominance of wheat segments at the western-southern part of the plain is due to the higher availability of water in a cooler climate, thus allowing wheat’s root system to proliferate horizontally and vertically for water extraction, benefiting from the fact that the water table is relatively shallow. Contrariwise, at the upper part of the plain, farmers generally prefer to cultivate barley or other crops that do not require any supplementary irrigation during the winter season.

To assess whether the change in areas between 2016 and 2017 was significant or error related, we followed the approach proposed by Olofsson et al. [38]. The approach relies basically on accuracy assessment sample data, in addition to the area proportions of each class, to eventually estimate the area of classified classes ± the standard error, with 95% confidence of interval. Referring to the areas of each year (Table 7), it appears that the wheat areas have significantly decreased between 2016 and 2017.

The decrease in areas is basically related to the rotation system (i.e., simple potato-wheat rotation) applied by most farmers in the plain. Thus, we expect an increase of these areas in 2018. Nevertheless, a change in the subsidy policy could discourage farmers from cultivating wheat in the future and could also be a reason behind the decrease in wheat cultivated area from 2016 through 2017. Actually, since 2016, the government has stopped purchasing the wheat production, instead, the Ministry of Economy (MoE) subsidizes farmers only by cultivated area with relatively small monetary amounts (800 USD/hectare), which barely cover the cultivation costs. The other constraint that farmers are facing is that Syrian borders are closed due to the ongoing Syrian civil war, which prevents them from exporting their production. While on the other hand, the Lebanese government is still importing wheat grains without any pre-consideration of the market needs leading to a fully saturated local market. For this reason, the deterioration of the unsold wheat production is never encouraging farmers to grow wheat throughout the coming years.

A comparison of the wheat areas obtained by *SEWMA* to those estimated by the Lebanese government (Table 7) illustrates a similarity in the obtained numbers. To be more specific, the government’s estimations rely on wheat farmers who declare that they cultivated wheat to benefit from the subsidy program, which is followed by field inspections. It is important to note that some wheat farmers do not give notice to the government, thus are not included in the governmental statistics. The difference between the areas estimated by *SEWMA* and those reported by the Lebanese government could be caused by several technical and human-related errors. First, discriminating triticale from wheat lands visually is generally difficult, even for specialized personnel. Second, fake reports could be submitted by farmers claiming that they have cultivated wheat, coupled with an impossibility of the corresponding teams to access their lands for field verification. Third, the estimations have started in 2016, thus, many farmers in that year have faced complications in submitting applications regarding their cultivated areas.

Although it is never recommended to avoid rotation, there is a number of plots that witnessed wheat cultivation in two consecutive years (2016 and 2017) (Figure 9). Because other crops are not supported (e.g., potato, vegetables and legumes), poor farmers who rent lands in order to maintain their livelihood tend to avoid the risk of growing other crops and keep cultivating wheat in monoculture despite the risk of soil borne diseases, knowing in advance that they will be subsidized by the government. 

The proposed method has allowed the mapping of winter wheat throughout 2016 and 2017. The outcome has proved that year-to-year transfer of knowledge is possible, if the evolution of a certain crop is well understood. Nevertheless, discriminating winter wheat among other winter cereal crops (i.e., barley and triticale) is doable using remotely-sensed data, in addition to ground observations.

### 5.5. Strengths, Limitations and Future Directions

The proposed method, Simple and Effective Wheat Mapping Approach (*SEWMA*), has proven to be successful in predicting wheat spatial distribution in the Bekaa plain of Lebanon for the years 2016 and 2017. 

*SEWMA* appears to have several strong points; (1) it only requires limited number of satellite imageries datasets in one single season for executing the classification, an option highly crucial in a developing country such as Lebanon; (2) it discriminates wheat from other similar winter cereals (i.e., barley and triticale) with only few field campaigns required; (3) it produces accurate (87%) early outputs in an automated way, thus saving resources and time.

In the same context, application of *SEWMA* is technically simple and easy to implement. However, it is site dependent and some requirements have to be met. For instance, replicating *SEWMA* in different regions may be affected by climate, farming conditions, agricultural practices and crop calendar. Concerning this, avoiding field visits in a new study site may result in critical drawbacks and unsatisfactory results.

Distinguishing winter wheat from barley and triticale could not be well achieved if the key phenological dates were not well known, particularly anthesis. In addition, irrigation practices were very important to deriving the conditions applied in the second phase of *SEWMA*. As previously mentioned (Section 5.3), wheat plots that are not irrigated due to shortage in water, could be susceptible to elimination, in addition to barley plots, during the second phase of *SEWMA*.

As reported in Sentinel-2 data quality report in 2018 [54], Sentinel-2A images before 15 June 2016 stem registration errors, due to three main contributors: (a) dynamic vibrations residuals mainly related to on-board oscillations; (b) static LOS calibration residuals; and (c) correlation noise and outliers. Thus, several previous studies [55,56,57] have shown a mis-registration between multi-temporal Sentinel-2A images from the same and different orbits for images acquired in 2016. To tackle the issue, we tried to visually investigate that matter by using the “chessboard” approach proposed by Shakun et al. [56] as well as the qualitative visual registration assessment described in [58]. In addition, an open source software based on Yan et al. [58] was used to quantify the occurred shifting in the x and y directions. The average mis-registration on the whole Sentinel-2 tile between 2016 and 2017 was around 0.068 ± 0.13 × 10 m in the x direction and 0.128 ± 0.263 × 10 m in the y direction. When quantifying the mis-registration on our study site, the shifting decreased to an average of 0.028 ± 0.1 × 10 m in the x direction and 0.034 ± 0.1 × 10 m in the y direction. It could be related to the location of our study area at the center of the Sentinel 2A tile (Figure 1) and far from the swath edges [55]. For future studies, an open source software [58,59] designed for mis-registration quantifications and corrections is recommended. These approaches are particularly needed when combining different sensors such as Landsat 8 and Sentinel-2 datasets.

As cloudy images are always a drawback when working with optical satellites, the availability of cloud-free datasets, or further pre-processing, is always recommended. Several algorithms have been proposed (e.g., Mean Attribute, Most Common Attribute value and k-nearest neighbor imputation) to fill the data lost by cloud removal [60]. The decision on whether to apply those algorithms, and which to choose amongst, is highly dependent on climatic and environmental conditions, as well as the purpose of use.

Furthermore, since Sentinel-2B was not launched before March, 2017, we could not benefit from its data for our study, otherwise, temporal resolution could be maximized (5-days) and more images could have been available. As other previous studies have proven, the usage of both optical and radar images would improve the classification, especially when pilot areas are covered with clouds [18,19]. The performance of *SEWMA* was tested on the Bekaa plain of Lebanon, which is a semi-arid climatic region. Hence, for future studies, including other climatic regions and enlarging the sampling data would generate better outputs. However, with a total accuracy of 87%, our proposed approach could be implemented across the Bekaa region and in similar climatic areas.

## 6. Conclusions

A novel wheat classification tree-like approach was presented in this study. Combining remote sensing with field observations allowed us to classify wheat throughout 2016 and 2017 four to six weeks prior to harvest which is highly important for any country with subsidy system. Moreover, the proposed approach, surnamed *SEWMA*, showed high accuracy in identifying wheat segments (87% in 2016 and 82.6% in 2017) even in climatically different years and with the existence of several crops with similar NDVI yearly profiles.

Wheat area decreased from 11,063 ± 1309 ha in 2016 to 7605 ± 1184 in 2017 due to different reasons including; (1) agricultural practices; (2) corrupt subsidy policies; (3) the Syrian war and (4) marketing policies. As for the spatial distribution of wheat in the area of study, we found that wheat cultivations were denser in areas with more available water and shallower water table (i.e., south-west Bekaa plain) to secure supplemental irrigation. 

Increasing the sustainability of water use and improved water productivity are some of the very essential goals of the Sustainable Development Goals (SDG). *SEWMA* in this sense plays a very effective role as its output allows forecasting the areas cultivated to allow controlling and sustainably managing wheat crops in food insecure countries. *SEWMA* is then an important tool that can be recommended for the monitoring of cultivated areas and assessment of expected yield by decision makers, food producers and trade managers, which could be integrated within the national and regional agricultural financial support systems.

## Figures and Tables

**Table 1:** Day of Year (DOY) of Sentinel-2 images used for both 2016 and 2017 cropping seasons.

Sentinel-2 Image Number	1	2	3	4	5	6	7	8
2016 DOY	17	47	67	87	97	107	117	137
2017 DOY	11	41	51	71	101	111	131	151

**Table 2:** Number of segmented plots visited per cultivations in 2016 and 2017.

Crop	2016	2017
Wheat	216	348
Barley	59	13
Triticale	64	17
Spring potato	111	117
Spring vegetables	14	20
Fruit trees	157	190
Vineyards	29	33
Alfalfa	11	23
Bare soil	7	8
Total	668	769

**Table 3:** Slope (a) and interception (b) deduced from the already produced linear equations.

Date Year	1	2	3	4	5	6	7	8
2016	DOY 17	DOY 47 a = 1.211 b = 0.111	DOY 67 a = 0.670 b = 0.492	DOY 87 a = 0.459 b = 0.516	DOY 97 a = 0.673 b = 0.287	DOY 107 a = 0.442 b = 0.566	DOY 117 a = 1.055 b = 0.077	DOY 137 a = 1.615 b = −0.773
2017	DOY 11	DOY 41 a = 0.724 b = 0.138	DOY 51 a = 1.042 b = 0.008	DOY 71 a = 0.893 b = 0.296	DOY 101 a = 0.268 b = 0.464	DOY 111 a = 0.759 b = 0.408	DOY 131 a = 1.041 b = 0.012	DOY 151 a = 1.426 b = −0.674

**Table 4:** Overall accuracies of wheat mapping using the three thresholds tested.

Threshold *SEWMA*	μ + *1*σ	μ + *1.5*σ	μ + *2*σ
Trained by 2016 and validated by 2017	84.0%	87.0%	84.7%
Trained by 2017 and validated by 2016	80.4%	82.6%	79.2%

**Table 5:** Confusion matrix of 2016 wheat classification trained by 2017 data.

ClassValue	Not Wheat	Wheat	Total	User Accuracy
Not wheat	331	104	435	0.761
Wheat	17	244	261	0.935
Total	348	348	696	
Producer Accuracy	0.951	0.701		0.826

**Table 6:** Confusion matrix of 2017 wheat classification trained by 2016 data.

ClassValue	Not Wheat	Wheat	Total	User Accuracy
Not wheat	189	29	218	0.867
Wheat	27	187	214	0.874
Total	216	216	432	
Producer Accuracy	0.875	0.866		0.870

**Table 7:** Areas estimates of wheat cultivated plots in the study area for years 2016 and 2017 (According to Olofsson et al. [39]).

Year	Wheat Area Estimated from *SEWMA* (ha) (Error-Corrected Estimates)	Wheat Area by Lebanese Government (ha)
2016	11,063 ± 1309	9073.4
2017	7605 ± 1184	7877.8
